# Mifepristone–Misoprostol Versus Misoprostol Alone for Early Missed Miscarriage After ART and Spontaneously Conceived Pregnancies

**DOI:** 10.3390/jcm14176340

**Published:** 2025-09-08

**Authors:** Adi Dayan-Schwartz, Revital Vinitski, Haya Hassan, Ido Izhaki, Suzan Abd Elgani, Liron Kogan, Shira Baram, Firas Zbidat, Khadeje Seh, Noah Zafran, Ari Reiss, Ronit Beck-Fruchter

**Affiliations:** 1 Department of Obstetrics and Gynecology, Emek Medical Center, Afula 1834111, Israellironkogan@clalit.org.il (L.K.);; 2 Rappaport Faculty of Medicine, Technion-Israel Institute of Technology, Haifa 3200003, Israel; 3 Department of Evolutionary and Environmental Biology, University of Haifa, Haifa 3103301, Israel; 4 Hadassah Medical Center, Faculty of Medicine, Hebrew University of Jerusalem, Jerusalem 9112001, Israel

**Keywords:** missed miscarriage, missed abortion, mifepristone, misoprostol, assisted reproductive technology (ART)

## Abstract

**Background**: Missed miscarriage (MM) is a common first-trimester complication. Misoprostol alone achieves moderate success, while combination therapy with mifepristone improves outcomes in spontaneous pregnancies. Evidence in assisted reproductive technology (ART) pregnancies is scarce. We evaluated whether combined mifepristone–misoprostol improves outcomes in ART pregnancies compared with misoprostol alone and compared results with spontaneously conceived (SC) pregnancies. **Methods**: This retrospective matched cohort study was conducted at a single center (2017–2024). ART pregnancies were matched 1:2 with SC pregnancies by maternal age. Patients received misoprostol alone or 200 mg mifepristone followed 48 h later by misoprostol. The primary outcome was treatment success, defined as complete uterine evacuation without repeat misoprostol or surgery. Secondary outcomes included emergency visits, surgical procedures, and ART-related predictors. Subgroup analyses were performed by ART protocol. **Results**: Among 307 patients (94 ART, 213 SC), combined therapy yielded higher success than misoprostol alone in SC (84% vs. 71%, *p* = 0.023) and ART pregnancies (95% vs. 80%, *p* = 0.035). In hormonally supported frozen embryo transfer (HRT-FET) cycles, success was 100% with combined therapy versus 80% with misoprostol alone. **Conclusions**: Combined mifepristone–misoprostol is more effective than misoprostol alone, with particularly high success in HRT-FET cycles.

## 1. Introduction

First trimester miscarriage occurs in about 15% of all recognized pregnancies and in approximately 30% of all pregnancies [1,2]. The rate of miscarriage after assisted reproductive technology (ART) is slightly higher than that of pregnancies conceived spontaneously, most likely due to older maternal age and more meticulous follow-up [3,4]. The incidence of missed miscarriage (MM) among all miscarriages has increased to 38.8% in recent years [1]. MM is diagnosed when a non-viable pregnancy is identified during the first trimester of gestation, with the retention of all pregnancy tissue inside the uterus [5].

MM can be managed in three ways: expectant management, medical, or surgical [6].

Since the 1980s, the primary medical treatment for MM uterine evacuation was a single dose of misoprostol (prostaglandin E1 analogue) [7]. While studies on misoprostol combined with mifepristone for MM and termination of pregnancy in the first trimester were conducted from the late 1980s [8,9], its use was only approved by the United States Food and Drug Administration for the induction of abortion in 2003 [10].

In recent years, medical management of early pregnancy loss has gained increasing acceptance, particularly with the introduction of dual therapy combining mifepristone and misoprostol, which has demonstrated higher effectiveness and reduced need for surgical intervention compared with misoprostol alone [11].

Mifepristone is a progesterone receptor antagonist that primes the myometrium before prostaglandin exposure in MM. Recent studies, including a large 2024 cohort, have confirmed the superiority of the combination over misoprostol alone, with significantly lower rates of treatment failure [5,6,11,12]. Data regarding mifepristone–misoprostol treatment for MM in ART-conceived pregnancies is scarce [3,13,14].

The ART pregnancy population and pregnancy characteristics differ in many ways from those of spontaneously conceived (SC) pregnancies. One distinction is the difference in progesterone levels between the groups, as ART pregnancies receive exogenous progesterone in fresh cycles or both estrogen and progesterone in hormone replacement therapy frozen embryo transfer (HRT-FET) cycles. This could affect the effectiveness of the combined treatment, which includes a progesterone receptor antagonist.

In the present study, we focused on ART pregnancies achieved through fresh embryo transfer, natural cycle frozen embryo transfer, and HRT-FET. Oocyte donation cycles and pregnancies following ovulation induction were excluded, as they constitute distinct populations with different hormonal characteristics. Given that ART outcomes are influenced by both ovarian stimulation and luteal support regimens, baseline endocrine parameters such as FSH, estradiol, and progesterone were collected to provide context on the hormonal milieu of ART patients and to assess whether these factors might influence the efficacy of medical management.

Our aim was to compare the success rates of combined mifepristone and misoprostol versus misoprostol alone for uterine evacuation in patients with MM from ART cycles and to compare these outcomes with those of spontaneously conceived pregnancies.

## 2. Materials and Methods

This was a retrospective matched cohort study conducted on data collected between 2017 and 2024 at Emek Medical Center, Israel. The study received approval from the Institutional Review Board (IRB #EMC-0047-23).

### 2.1. Patient Eligibility and Diagnosis of Missed Miscarriage

We included patients with first-trimester MM. Diagnosis was based on internationally recognized ultrasound sonographic criteria for non-viability [15]. Specifically, MM was diagnosed when a transvaginal scan demonstrated a crown–rump length (CRL) of at least 7 mm without embryonic cardiac activity or when the mean gestational sac diameter was 25 mm or greater with no visible embryo. In addition, pregnancies were considered non-viable if embryonic cardiac activity was absent on a follow-up scan performed at least 11 days after a gestational sac with a yolk sac was first observed or at least 14 days after an empty gestational sac was identified. These criteria are endorsed by both the American College of Obstetricians and Gynecologists (ACOG) and the Royal College of Obstetricians and Gynaecologists (RCOG) to ensure accurate diagnosis and minimize the risk of intervening in a potentially viable pregnancy. All ultrasound examinations were performed using high-resolution transvaginal probes by an obstetric physician, and in ambiguous cases, repeat scanning was performed by a senior attending physician for confirmation.

### 2.2. Treatment Protocols

Medical management of MM was standardized in our department. Until 2019, the routine protocol consisted of a single dose of 800 μg vaginal misoprostol. From 2020 onward, the protocol was revised to include 200 mg oral mifepristone, administered once, followed 48 h later by 800 μg oral misoprostol. Medical uterine evacuation was offered to patients with gestational age ≤ 9 weeks + 6 days, confirmed by CRL measurement. Patients were discharged with clear instructions and advised to attend follow-up evaluation within 2–3 weeks, which included both clinical review and repeat ultrasound to confirm completion of uterine evacuation.

### 2.3. Study Cohort and Control Group

The study cohort included all ART-conceived pregnancies treated medically for MM during the study period. Each ART-conceived pregnancy was matched to two spontaneously conceived (SC) pregnancies based on maternal age, ensuring comparability between groups. Exclusion criteria comprised incomplete miscarriage, viable pregnancies, pregnancy of unknown location, patients who underwent primary surgical or expectant management, incorrect drug dosage or administration route, oocyte donation cycles, and ART pregnancies with incomplete treatment records (e.g., cycles initiated at another institution).

### 2.4. Data Collection and ART Cycle Classification

Patients were identified through electronic medical records using the ICD-9 code 632 (“missed abortion”). Fertility clinic records corresponding to each ART case were reviewed to extract detailed data. Information collected included the etiology of infertility, ART type, ovarian stimulation protocol, baseline FSH, serum estradiol and progesterone levels, pre-transfer endometrial thickness, number of oocytes retrieved, and number and stage of embryos transferred. Cleavage-stage embryos (day 2–3) were used in all ART cycles during the study period.

ART cycles were stratified into three categories:

1. Fresh embryo transfer (Fresh ET): Embryo transfer performed in the same cycle as ovarian stimulation and oocyte retrieval.
2. HRT-FET: Endometrial preparation with exogenous estradiol, followed by progesterone supplementation. In this group, there is no corpus luteum, and all steroid hormones are supplied exogenously.
3. Natural cycle frozen embryo transfer (Natural FET): Transfers performed during spontaneous ovulatory cycles and with corpus luteum function preserved.

The hormonal support protocols used in each subgroup: In Fresh ET and Natural FET cycles, patients received vaginal micronized progesterone (progesterone vaginal tablet, Endometrin^®^) 100 mg twice daily, continued up to 8 weeks of gestational age (GA). In HRT-FET cycles, patients received oral estradiol hemihydrate (Estrofem^®^, Novo Nordisk, Bagsvaerd, Denmark) 6 mg daily, combined with vaginal micronized progesterone (Endometrin^®^, Ferring Pharmaceuticals, Parsippany, NJ, USA) 400 mg twice daily and intramuscular progesterone (Prontogest^®^, IBSA Pharma, Parsippany, NJ, USA) 50 mg every 3 days, continued up to 12 weeks of GA.

### 2.5. Outcome Definitions

The primary outcome was treatment success, defined as complete uterine evacuation without the need for surgical intervention (dilatation and curettage or hysteroscopy) and without administration of an additional misoprostol dose. Treatment failure was defined as the requirement for either repeat misoprostol, surgical evacuation, or hospital re-admission due to complications such as persistent bleeding or infection. Secondary outcomes included return visits to the emergency department, type of surgical intervention required, and identification of demographic or ART-related variables associated with treatment success.

### 2.6. Statistical Analysis

Normally distributed variables are presented as mean ± standard deviation (SD) and compared using the independent-samples *t*-test. Non-normally distributed variables are presented as median with interquartile range (IQR) and compared using the Mann–Whitney *U* test or the Kruskal–Wallis test as appropriate. Categorical variables were reported as absolute numbers and percentages and compared using the chi-square test or Fisher’s exact test as appropriate. Multivariate logistic regression analysis was performed to adjust for potential confounders and to identify independent predictors of treatment outcome. A *p*-value < 0.05 was considered statistically significant.

## 3. Results

**Figure 1:**
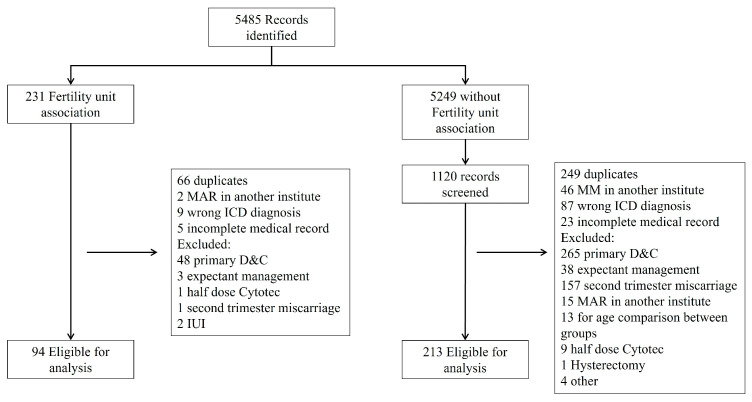
Study inclusion diagram.

Overall, 307 patients were included in the study as presented in Figure 1. Ninety-four patients (31%) conceived through ART, with fifty-one (54%) undergoing uterine evacuation treatment with misoprostol alone and forty-three (46%) receiving additional mifepristone. Two hundred and thirteen patients (69%) conceived spontaneously, with one hundred and seventeen (55%) treated with misoprostol alone and ninety-six (45%) receiving additional mifepristone. Demographic and obstetric variables are presented in Table 1. Maternal age did not differ significantly between the ART and SC groups due to predetermined matching. GA was significantly lower in the ART group compared to the SC group, while the CRL at diagnosis was similar in both groups. The SC group had higher gravidity, parity, and number of living children (*p* = 0.001). A multivariate logistic regression analysis of the statistically significant variables showed no statistical difference between the ART and SC groups.

Overall, no significant difference was found between the success rates of the SC and ART-conceived groups. However, there was a trend toward a higher success rate with the combined treatment in the ART group compared to the SC group (95.3% versus 84.3%, *p* = 0.093).

The success rate of the combined treatment was significantly better in both SC and ART-conceived groups (84% versus 71%, *p* = 0.023; 95% versus 80%, *p* = 0.035, respectively). Furthermore, there was a significantly higher rate of patients returning to the emergency room in the misoprostol alone groups, observed in both SC and ART-conceived cohorts (30% versus 13.5%, *p* = 0.005; 23.5% versus 7%, *p* = 0.046, respectively).

A higher proportion of patients in the misoprostol only treatment group required dilatation and curettage (D&C). The difference was statistically significant, being higher in the SC MM group compared to the combined treatment (15% versus 4%, *p* = 0.011), (Table 2). Thirty-three patients conceived via HRT-FET cycles and experienced missed miscarriages (MMs). The overall success rate was 87.9%. Among these patients, twenty were treated with misoprostol alone, while thirteen received additional mifepristone. The success rates for these groups showed a trend toward a higher success rate with the combined treatment (80% versus 100%, respectively, *p* = 0.085). ART treatment variables are presented in Table 3. Eleven patients conceived during natural FET cycles with a total success rate of 91%. The small sample size precludes comparison between the two treatment protocols (Table 4).

There was no statistical difference between the ART-conceived patient characteristics of the combined treatment group to the misoprostol alone treatment group. Infertility reason was not statistically different between treatment groups among the ART patients (Table 3). ART protocols and treatment characteristics were analyzed for impact on MM treatment success, with no statistically significant difference.

Demographic and obstetric variables were analyzed as predictors of treatment success, and, only in the ART group, low BMI emerged as a statistically significant independent predictor (OR 1.3, CI 1.05–1.8). BMI data was not available in the SC group.

## 4. Discussion

Our study demonstrates that sequential treatment with mifepristone followed by misoprostol for MM is better than misoprostol alone in both SC and ART patients. The combined treatment had a higher rate of complete uterine evacuation without additional intervention. In the SC pregnancy population, our findings are consistent with previous studies [5,6,12]. Chu et al. (2020) [5] published a large multicenter, randomized, double-blind, placebo-controlled trial showing the advantage of the combined treatment in MM completion within 7 days and fewer incidences of surgical intervention compared with misoprostol alone.

Recently, a few studies have addressed the issue of medically induced uterine evacuation for MM in the ART patient population [3,13]. Esposito et al. published a case series of nine patients who underwent in vitro fertilization followed by fresh or FET. All nine received 200 mg of mifepristone 24 h before 800 mg of misoprostol. Eight subjects had successful treatment and one required uterine aspiration.

Colleselli-Türtscher et al. compared SC to ART-conceived MM with combined treatment with 831 SC pregnancies (89%) and 99 ART-conceived pregnancies (11%). An additional 400 mcg misoprostol was administered to patients with no vaginal bleeding after 5–6 h, based on the treating physicians’ discretion. All patients had a follow-up assessment one week after treatment for the necessity of additional intervention. Treatment success was defined as no need for further surgical intervention, even with the need for additional medical intervention. Patients in the ART group were significantly older and had lower GA at treatment according to the last menstrual period (LMP). The overall success rate of medical treatment of MM was 89% (825/930), with no statistically significant difference according to the mode of conception. There were no differences in success rate between pregnancies resulting from fresh (89%) and frozen (89%) embryo transfers. Only sonographic GA was a statistically significant independent predictor of success. The ART characteristics were not included in the paper, there was no control group of misoprostol alone treatment, and the groups were not stratified by patients’ age.

A recent retrospective cohort by Gluck et al. (2024) [14] evaluated medical management of early pregnancy loss in 775 patients, comparing IVF and spontaneously conceived pregnancies. They found no difference in overall treatment failure between groups, although IVF pregnancies had a significantly lower risk of emergency dilation and curettage. Importantly, their cohort was treated exclusively with a misoprostol only regimen and they did not stratify assisted conception pregnancies by protocol. In contrast, our study provides novel insights by analyzing ART subgroups, including HRT-FET, where corpus luteum activity is absent, and by evaluating the combined mifepristone–misoprostol regimen, which has become the preferred treatment for missed miscarriage.

In our study, the overall success rate, when analyzing the same treatment type, was higher in the ART group compared to the SC groups, without statistical significance. We did observe a trend of higher success rate for the combined treatment in the ART group compared to the combined treatment of the SC group. The lower GA despite similar CRL at uterine evacuation in the ART group may be attributed to their meticulous follow-up and diagnostic procedures. The higher success rates in the ART groups could be attributed to earlier diagnosis. However, the similar CRL between the groups challenges this assumption.

The HRT-FET subgroup, in which endometrial preparation relies entirely on exogenous estradiol and progesterone, may respond differently to mifepristone. Unlike fresh or natural cycle FET, where the corpus luteum provides endogenous steroid hormones and vasoactive mediators such as relaxin and vascular endothelial growth factor that support early placentation [16,17], HRT-FET cycles lack these endogenous contributions. This creates a unique endocrine environment that is wholly dependent on pharmacologic supplementation. When missed miscarriage is diagnosed and luteal support is withdrawn, the abrupt and complete withdrawal of estradiol and progesterone may synergize with the progesterone receptor blockade of mifepristone, accelerating endometrial breakdown and uterine evacuation. By contrast, in cycles with an active corpus luteum, residual endogenous hormone production may counteract or delay these processes. This biological difference could explain why combined therapy shows particularly high efficacy in the HRT-FET subgroup and underscores the need to explore mechanistic pathways linking corpus luteum function, luteal support regimens, and the response to medical management of miscarriage.

It should also be noted that hormonal support regimens differed between ART subgroups, which may have influenced treatment outcomes. In particular, discontinuation of luteal support at the time of miscarriage diagnosis could have a differential impact, especially in HRT-FET cycles where all steroid hormones are exogenously supplied. This warrants further investigation in future studies.

We observed a 20% success difference between the treatments in the HRT-FET subgroup with the advantage of the combined treatment. The small group size of the FET subgroup may explain the non-significant differences. Furthermore, the natural cycle FET with the presence of CL had a success rate of 89% in the combined treatment group, which is lower than the 100% success rate of the HRT-FET subgroup (Table 4).

The strengths of this study are the strict selection of patients for each group with the same treatment type and patient age stratification, while additional misoprostol intervention was regarded as treatment failure. The ART groups had detailed analysis of the ART protocol characteristics that could impact treatment success rate, including FET with natural menstrual cycles and HRT-FET. The study had a control group of misoprostol alone for both SC and ART-conceived groups. We had thorough follow-up for treatment outcomes that included the patients’ primary care visits, visits to other hospitals, and even late interventions with hysteroscopic evacuation of retained products of pregnancy.

Limitations of this study include those inherent to any retrospective study. Additionally, we compared mifepristone–misoprostol treatment to misoprostol alone while the misoprostol dosage did not differ at 800 mcg; the route of administration was changed from vaginal to oral. Vaginal, sublingual, or buccal administration is an accepted method of medication administration for MM [18]. Although the route of administration was changed, we compared the same route of administration and dosage for ART-conceived to SC combined treatment and misoprostol alone for those two groups as well. Our exclusion criteria did not address somatic diseases, and outcomes were not stratified by comorbidities, which may limit generalizability. A further limitation is the external validity of our findings, as the study was conducted at a single center in Israel with a relatively small sample size (307 patients), and the results may not fully generalize to other geographic regions or healthcare systems.

## 5. Conclusions

In this large retrospective matched cohort, we confirm that combined mifepristone and misoprostol treatment is more effective than misoprostol alone for medical management of missed miscarriage, both in spontaneously conceived and ART-conceived pregnancies. The novelty of our study lies in the detailed analysis of ART subgroups, suggesting that HRT-FET cycles may derive particular benefit from mifepristone due to their unique endocrine environment lacking corpus luteum activity.

While our findings are strengthened by strict case selection, detailed ART protocol data, and robust outcome definitions, they are limited by the retrospective design, variation in misoprostol administration route across the study period, and relatively small subgroup sizes.

Future research should include prospective multicenter randomized trials stratified by ART protocol, especially differentiating HRT-FET from natural cycle FET, to confirm whether corpus-luteum-dependent hormonal differences influence treatment success. Mechanistic studies examining the role of vasoactive substances secreted by the corpus luteum could further clarify the biological interaction with mifepristone.

Overall, our results support the routine use of mifepristone–misoprostol combination therapy for missed miscarriage and provide an important foundation for tailoring medical management in patients undergoing assisted reproduction.

## Figures and Tables

**Table 1:** Demographic and obstetric variables of Study Group and Control Group.

Variable	Groups			
	Study Group (*n* = 94)		Control Group (*n* = 213) Total	
	Mifepristone + Misoprostol (*n* = 43)	Misoprostol Alone (*n* = 51)	Mifepristone + Misoprostol (*n* = 96)	Misoprostol Alone (*n* = 117)
Maternal age	37 (32.5–40)	36 (30–39)	37 (35–38)	35 (34–39)
Ethnicity (Jewish)	28 (65.1%)	**27 (53%)** ^1^	71 (74%)	**93 (79%)** ^1^
Smoking	**9 (21%)** ^2^	7 (14%)	**8 (8.3%)** ^2^	14 (12%)
**Gravidity** ^3^	2.6 ± 1.8	2.3 ± 1.5	4 ± 2	4 ± 1.8
**Parity** ^3^	0.6 ± 0.8	0.6 ± 0.8	2.2 ± 1.6	2.3 ± 1.4
Abortion	0.9 ± 1.3	0.7 ± 1.1	0.8 ± 1	0.7 ± 1
History of EP	**4 (9.3%)** ^4^	1 (2%)	**1 (1%)** ^4^	3 (2.5%)
CS	0.2 ± 0.5	0.2 ± 0.6	0.4 ± 1	0.3 ± 0.7
**Living children** ^3^	0.6 ± 0.8	0.6 ± 1.0	2.2 ± 1.7	2.2 ± 1.4
History of pre-term delivery	0	2 (4%)	2 (2%)	1 (0.9%)
**GA at abortion (days)** ^3^	58 (53.5–61.5)	60 (54–64)	64 (58.5–70)	63 (58.8–69)
CRL at abortion (days)	42 (35–43.5)	42 (35–45.5)	42 (35–48.2)	42 (35–47)
BMI	27.6 ± 5.3	26.0 ± 4.8	NA	NA

Results are presented as mean ± SD or *n* (%) when normally distributed and as median (IQR) when distributions are non-normal. Bold values indicate statistically significant differences between groups (*p* < 0.05). No statistical difference between combined and Misoprostol alone treatment groups (in both SC and ART groups). ^1^ *p*-Value = 0.001 (ART Misoprostol alone vs. Spontaneously Conceived Misoprostol alone group). ^2^ *p*-Value = 0.05 (ART Mifepristone + Misoprostol vs. Spontaneously Conceived Mifepristone + Misoprostol group). ^3^ *p*-Value = 0.001 between study and control groups for both combined and Misoprostol alone treatments. No statistical difference on multivariate logistic regression. ^4^ *p*-Value = 0.048 (ART Mifepristone + Misoprostol vs. Spontaneously Conceived Mifepristone + Misoprostol group).

**Table 2:** Outcomes By treatment and ART vs. Spontaneous.

Variable	Study Group (*n* = 94)		Control Group (*n* = 213)	
	Mifepristone + Misoprostol (*n* = 43)	Misoprostol Alone (*n* = 51) Total	Mifepristone + Misoprostol (*n* = 96)	Misoprostol Alone (*n* = 117)
Treatment success ^1^	**41 (95%)**	**41 (80%)**	**81 (84%)**	**83 (71%)**
Return to the ER ^2^	**3 (7%)**	**12 (23.5%)**	**13 (13.5%)**	**35 (30%)**
Cytotec second dose	0	2 (4%)	4 (4%)	9 (8%)
D&C ^3^	**1 (2.3%)**	**4 (8%)**	**4 (4%)**	**18 (15%)**
Hysteroscopy	2 (4.6%)	4 (8%)	8 (8.3%) ^4^	9 (8%)
Surgical intervention ^5^	2 (4.6%)	7 (14%)	12 (12.5%)	25 (21%)

Results are presented as mean ± SD or *n* (%) when appropriate. Bold values indicate statistically significant differences between groups (*p* < 0.05). ^1^ *p*-Value = 0.093 between study group and control group combined treatment; *p*-Value = 0.035 between the two types of treatment in the study group; *p*-Value = 0.023 between the two types of treatment in the control group. ^2^ *p*-Value = 0.046 between the two types of treatment in the study group; *p*-Value = 0.005 between the two types of treatment in the control group. ^3^ *p*-Value = 0.011 between the two types of treatment in the control group. ^4^ Surgical Intervention = D&C or Hysteroscopy. ^5^ One patient in the control group had failed treatment with hysteroscopy for retained products of pregnancy, although there was no evidence of it in the operation.

**Table 3:** ART group variables.

Variable	Mifepristone + Misoprostol (*n* = 43)	Misoprostol Alone (*n* = 51)
Infertility diagnosis		
Male factor	18 (42%)	27 (52.9%)
Polycystic ovary syndrome	5 (11.6%)	9 (17.6%)
Mechanical factor	12 (28%)	15 (29.4%)
Unexplained	7 (16.3%)	7 (13.7%)
FSH	7 ± 2.6	7 ± 2.7
Fresh embryo transfer	21 (49%)	27 (53%)
HRT-FET	13 (30.2%)	20 (39.2%)
Natural cycle FET	9 (21%)	2 (4%)
Endometrium thickness	9.8 ± 2.3	9.8 ± 2.8
Embryo age (days)	2.6 ± 1.06	2.5 ± 0.5
Oocyte number	8.4 ± 5.2	8.6 ± 4.3
Number of fresh embryos returned	1.5 ± 0.5	1.6 ± 0.5
Number of frozen embryos returned	1.5 ± 0.5	1.7 ± 0.6
HCG (mIU/mL)	210.7 ± 254.2	296 ± 655.8
Progesterone2 (ng/mL)	28.6 ±19.2	32.4 ± 21.6
Estradiol2 (pg/mL)	590.7 ± 385.5	525.8 ± 526.9
Number of gestational sacs	1.1 ± 0.5	1.1 ± 0.4
No embryonic pulse on follow-up ^1^	10 (62.5%)	17 (43.6%)

Results are presented as mean ± SD or *n* (%) when appropriate. ^1^ Only one case of 2 pulses (in the Misoprostol alone group). Combined treatment group *N* = 16.

**Table 4:** Treatment success by ART subgroups.

Subgroup	Mifepristone + Misoprostol Successful Treatment	Misoprostol Alone Successful Treatment
Fresh embryo transfer (*n* = 50)	20 (95.2%)	23 (79.3%)
HRT-FET ^1^ (*n* = 33)	13 (100%)	16 (80%)
Natural cycle FET (*n* = 11)	8 (89%)	2 (100%)
Total (*n* = 94)	41 (95.3%)	41 (80.4%)

Results are presented as mean ± SD or *n* (%) when appropriate. ^1^ *p*-Value = 0.085.

## Data Availability

The data from this study is available from the corresponding author upon a reasonable request and following approval of the Institutional Review Board.

## Abbreviations

The following abbreviations are used in this manuscript:

MM	Missed Miscarriage: A first-trimester pregnancy loss where the embryo or fetus has died but the body has not yet expelled the pregnancy tissue.
ART	Assisted Reproductive Technology: Medical procedures used to achieve pregnancy, such as in vitro fertilization (IVF).
MAR	Medically Assisted Reproduction: Medical procedures used to achieve pregnancy, including all fertility treatments, from IVF to ovulation induction.
SC	Spontaneously Conceived Pregnancies: Pregnancies that occur without medical assistance or ART.
FET	Frozen Embryo Transfer: A procedure in which a previously frozen embryo is thawed and transferred to the uterus.
HRT-FET	Hormone Replacement Therapy Frozen Embryo Transfer: A frozen embryo transfer cycle in which the endometrium is prepared with exogenous hormone replacement therapy (estrogen and progesterone).
CL	Corpus Luteum: A temporary endocrine structure involved in the production of progesterone, critical for maintaining early pregnancy.
D&C	Dilatation and Curettage: A surgical procedure to remove tissue from the uterus, often used after failed medical management of miscarriage.
CRL	Crown–Rump Length: An ultrasound measurement used to assess gestational age in early pregnancy.
ICD	International Classification of Diseases: Global coding system for diseases and health conditions.
BMI	Body mass index
CS	Cesarean section
EP	Ectopic pregnancy
GA	Gestational age
FSH	Follicle-stimulating hormone
HCG	Human chorionic gonadotropin
